# The identification of hub-methylated differentially expressed genes in osteoarthritis patients is based on epigenomic and transcriptomic data

**DOI:** 10.3389/fmed.2023.1219830

**Published:** 2023-07-03

**Authors:** Zhen-Chen Chu, Ting Cong, Jian-Yu Zhao, Jian Zhang, Zhi-Yuan Lou, Yang Gao, Xin Tang

**Affiliations:** ^1^Department of Orthopedics, First Affiliated Hospital of Dalian Medical University, Dalian, Liaoning, China; ^2^Dalian Medical University, Dalian, Liaoning, China; ^3^Department of Anesthesiology, Second Affiliated Hospital of Dalian Medical University, Dalian, Liaoning, China

**Keywords:** osteoarthritis, epigenome, transcriptome, WGCNA, bioinformatics analysis

## Abstract

**Introduction:**

Osteoarthritis (OA) refers to a commonly seen degenerative joint disorder and a major global public health burden. According to the existing literature, osteoarthritis is related to epigenetic changes, which are important for diagnosing and treating the disease early. Through early targeted treatment, costly treatments and poor prognosis caused by advanced osteoarthritis can be avoided.

**Methods:**

This study combined gene differential expression analysis and weighted gene co-expression network analysis (WGCNA) of the transcriptome with epigenome microarray data to discover the hub gene of OA. We obtained 2 microarray datasets (GSE114007, GSE73626) in Gene Expression Omnibus (GEO). The R software was utilized for identifying differentially expressed genes (DEGs) and differentially methylated genes (DMGs). By using WGCNA to analyze the relationships between modules and phenotypes, it was discovered that the blue module (MEBlue) has the strongest phenotypic connection with OA (cor = 0.92, p = 4e-16). The hub genes for OA, also known as the hub methylated differentially expressed genes, were identified by matching the MEblue module to differentially methylated differentially expressed genes. Furthermore, this study used Gene set variation analysis (GSVA) to identify specific signal pathways associated with hub genes. qRT-PCR and western blotting assays were used to confirm the expression levels of the hub genes in OA patients and healthy controls.

**Results:**

Three hub genes were discovered: HTRA1, P2RY6, and RCAN1. GSVA analysis showed that high HTRA1 expression was mainly enriched in epithelial-mesenchymal transition and apical junction; high expression of P2RY6 was mainly enriched in the peroxisome, coagulation, and epithelial-mesenchymal transition; and high expression of RCAN1 was mainly enriched in epithelial-mesenchymal-transition, TGF-β-signaling, and glycolysis. The results of the RT-qPCR and WB assay were consistent with the findings.

**Discussion:**

The three genes tested may cause articular cartilage degeneration by inducing chondrocyte hypertrophy, regulating extracellular matrix accumulation, and improving macrophage pro-inflammatory response, resulting in the onset and progression of osteoarthritis. They can provide new ideas for targeted treatment of osteoarthritis.

## 1. Introduction

Osteoarthritis (OA) has been considered a commonly seen form of chronic degenerative joint disorder, with the characteristic of osteophyte formation, subchondral bone hyperplasia, cartilage deterioration, and narrowing of the joint space (1). OA affects about 250 million people worldwide (2). In the US, over 47 million people are considered to have OA, and the number is predicted to increase to 67 million by 2030 (or 25% of all adults) (3). There are about 3 million new diagnosed cases each year, especially among the elderly (4). As reported by World Health Organization (WHO), nearly 9.6% of males, together with 18% of females aged 60 years old, develop OA, among whom, 25% develop a disability (5). Furthermore, chronic pain and impairment related to osteoarthritis (OA) could promote depression, suicide, and anxiety (6). Side reactions in gastrointestinal and cardiovascular systems are the result of the long-run non-steroidal anti-inflammatory drugs (NSAID) application among OA cases (7, 8). Joint replacement surgery is an efficient method for treating advanced knee osteoarthritis; however, the procedure is expensive and fraught with postoperative complications (9, 10). Currently, OA can be predominantly diagnosed based on clinical symptoms and imaging; therefore, it is impossible to make an accurate early diagnosis (11). Consequently, there is a poor prognosis and ineffective treatments in the majority of patients. Although numerous risk factors are found to be related to OA, genetics and epigenetics have critical effects on its pathogenesis, which are considered hot areas for OA research (12). It is important to diagnose and treat OA early to improve the prognosis of the patient.

Epigenetics is the study of gene expression changes caused by non-gene sequence changes. DNA methylation, histone modification, chromosomal remodeling, and non-coding RNA are examples of epigenetic gene expression control. DNA methylation modification plays an important role in the regulation of gene expression in epigenetics (13). According to Imagawa et al., COL9A1 mRNA level markedly decreased, and six CpG sites on the COL9A1 promoter showed a strong hypermethylation level among OA chondrocytes. The administration of 5-Aza, a DNA methylation inhibitor, boosted the transcription of COL9A1 mRNA (14). Bioinformatics technology combined with epigenetics to study gene expression has been continuously optimized in recent years. For example, the MASQC algorithm, MTGIpick software, and recursive feature selection with random forest can reduce information redundancy while improving prediction efficiency and accuracy (15–17). Some visualization techniques can even show gene mutations, domains, and epitopes, and more detailed information about the disease, region, race, and related literature (18). Moreover, Moradi et al. (19) pointed out immune cell infiltration within joint tissues of OA containing CD8+ T cells, CD4+ T cells, CD16+ CD56+ NK cells and CD14+ macrophages. So, to comprehend the mechanism related to OA pathogenic mechanism and develop novel targets as well as signal pathways for immunotherapy, accurate biomarkers should be identified multidimensional to predict the pathogenesis of OA; thus, achieving multi-targeted treatment of OA.

This study explored epigenome and analyzed transcriptome microarray data. Transcriptome and methylated microarray datasets of OA were acquired in Gene Expression Omnibus (GEO) for detecting important genes associated with OA pathogenesis. To identify hub genes, this was combined with weighted gene co-expression network analysis (WGCNA). The functional enrichment of these three genes (*HTRA1*, *P2RY6*, and *RCAN1*) and their relationship with immune cells were analyzed synchronously. Furthermore, the hub genes were validated using qRT-PCR and Western blotting with samples of knee cartilage from patients with knee osteoarthritis and femoral head cartilage from healthy people. Therefore, the three hub genes discovered in the present work might be the candidate novel anti-OA targets.

## 2. Materials and methods

### 2.1. Datasets

**Figure 1:**
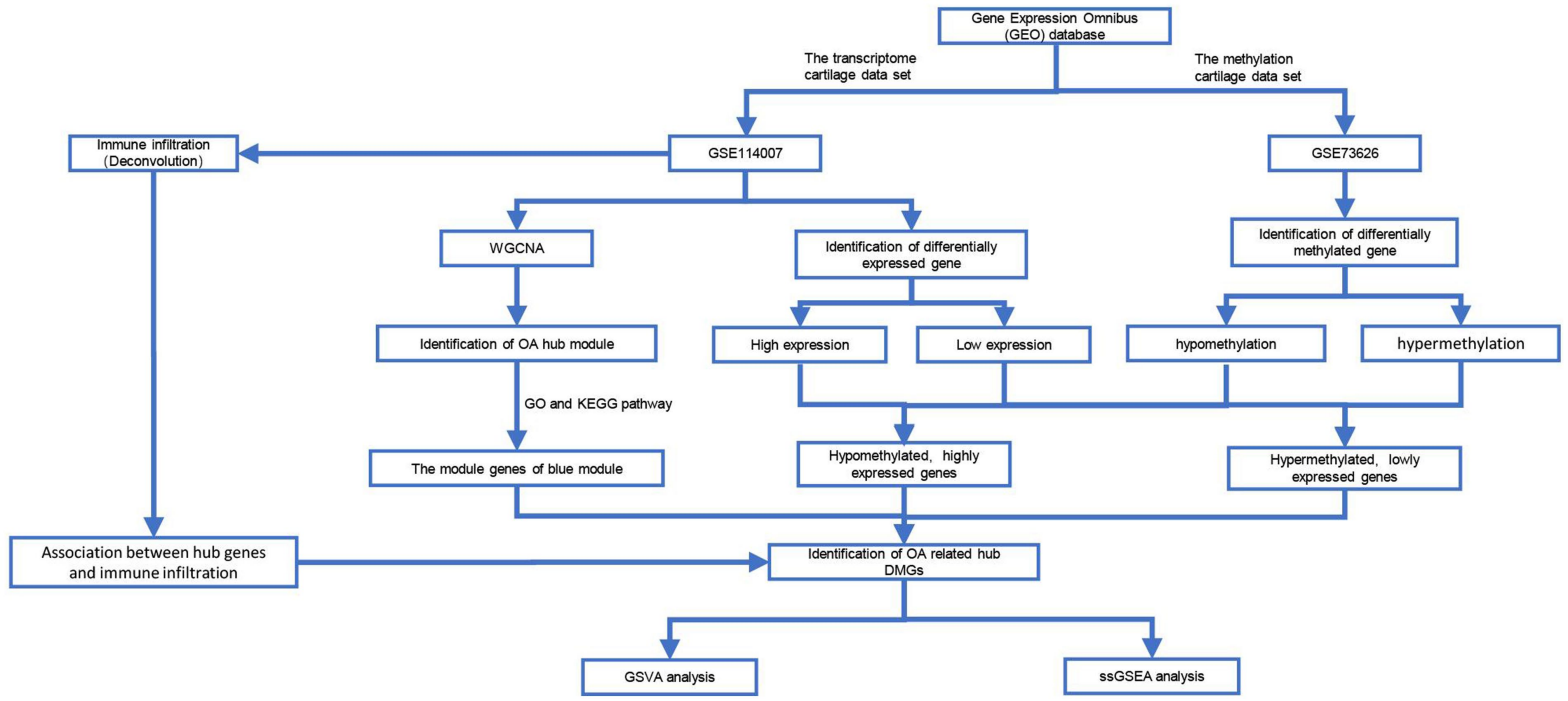
Data processing process in this work. GSVA, Gene Set Variation Analysis; WGCNA, Weighted Gene Co-expression Network Analysis; ssGSEA, Single-Sample Gene Set Enrichment Analysis.

DNA methylation, as an epigenetic control mechanism, regulates gene expression. DNA methylation has been shown to inhibit gene activity, whereas demethylation can induce gene reactivation and expression. It has an impact on a variety of biological processes and diseases. Epigenetic regulation is thought to play an important role in the onset and progression of OA. A comparison of methylation sites in DNA from OA cartilage and age-matched non-pathogenic chondrocytes was discovered using a genome-wide methylation analysis. Therefore, combining gene expression and DNA methylation analysis can provide new insights into the pathogenesis of OA. We selected 2 microarray datasets in the GEO database. The criteria for sample selection were: (1) samples from knee cartilage tissue from OA or healthy control, and (2) the number of samples >15. So the transcriptome (GSE114007) (20) and methylation (GSE73626) (21) cartilage data sets were chosen. The selected dataset (GSE114007) included the expression profiles of messenger RNA (mRNA) in 20 patients with osteoarthritis (OA) and 18 healthy individuals. Additionally, from the series matrix files of the methylation dataset (GSE73626), we acquired the DNA methylation profiles of 11 patients with osteoarthritis (OA) and 7 healthy individuals (see Figure 1).

### 2.2. DNA methylation together with transcriptome profiles

DNA methylation is essential for transcription knockdown and control of the gene level. Therefore, we retrieved the GSE114007 dataset in GEO of NCBI containing 38 sets of transcriptome data from the control (*n* = 18) and OA groups (*n* = 20). The Limma package in R software (limma_3.44.3) was used to detect differentially expressed genes (DEG) on *p* < 0.05 and |log FC| > 1 as significant thresholds.

The series matrix files of the methylation dataset (GSE73626) containing the DNA methylation profiles of healthy individuals (*n* = 7) and patients with OA (*n* = 11) could be obtained from the NCBI GEO public database. The locations of the differentially methylated genes (DMGs) were investigated using the ChAMP package of R software (ChAMP_2.16.2) and the following criteria: one group’s value should be <0.2, the other group’s value should be >0.3, and *p* < 0.05.

In this study, gene ontology (GO) analysis and Kyoto Encyclopedia of Genes and Genomes (KEGG) pathway analysis were performed on specific genes using the Metascape database for annotation and visualization.^1^ The significance levels were set to a min overlap of 3 and a *p* ≤ of 0.01.

### 2.3. Construction of the weighted gene co-expression network

For detecting co-expression modules as well as vital genes in the network and investigating the association of gene network with phenotype, this study attempted to build the co-expression network from the GSE114007 dataset with R software WGCNA package (WGCNA_1.71). A similarity matrix is constructed by calculating the correlation between all gene pairs and then ranking them according to the size of the variance to filter out the top 5,000 genes. A threshold value of 3 was selected as the suitable soft-threshold power β with the integration function (sft$powerEstimate) for WGCNA. To measure network connection, this work converted topological overlap matrix (TOM) from the weighted adjacency one. With the aim of building the clustering tree structure in the TOM matrix, the hierarchical clustering approach was adopted. Different gene modules were marked with different colors and symbolized by various branches of the cluster tree. Genes were classified according to their expression profiles; genes that showed close patterns were classified as one module. Meanwhile, thousands of genes were classified as various modules based on their expression patterns. The entire classification was built upon gene-weighted correlation coefficients.

### 2.4. Hub gene and module identification

The module-eigengenes (MEs) were subjected to principal component analysis (PCA), followed by Pearson correlation analysis of MEs with OA phenotypes to identify the hub module with the most significant correlations, and then genes in the hub module were matched with differentially methylated differentially expressed genes to screen out the hub genes associated with OA.

### 2.5. Patient and sample acquisition

Twelve knee cartilage tissues and 6 hip cartilage tissues were taken from 18 patients operated at the Department of Orthopedics, The First Affiliated Hospital of Dalian Medical University, between November 2020 and October 2022. Among them, 6 cases of hip cartilage in the normal group were taken from patients with femoral neck fractures, 3 cases of knee cartilage in the normal group were taken from patients with superior knee amputation, and 9 cases of primary knee osteoarthritis cartilage were taken from stage IV patients with knee osteoarthritis after total knee arthroplasty as the OA group. After the operation, the cartilage tissues were preserved under −80°C until analysis. The approval of this study was acquired from Ethics Committee of First Affiliated Hospital of Dalian Medical University (Number: PJ-KS-KY-2022–236).

### 2.6. Immune gene correlation

Immune-associated fibroblasts, extracellular matrix (ECM), immunological cells, inflammatory factors, growth factors, as well as unique physical and chemical properties comprise the majority of the immune microenvironment that have an impact on the diagnosis, prediction, and treatment of OA. Those underlying molecular mechanisms of hub genes regulating the development of disease were deeply analyzed by investigating hub gene relations with immune infiltration of that given dataset. Additionally, to identify degrees of immune cell infiltration of the samples, the single-sample gene set enrichment analysis (SSGSEA) (GSVA_1.36.3) was conducted, while the relations of gene expression with infiltrating immune cell abundances were analyzed based on Spearman’s correlation analysis.

### 2.7. Gene set variation analysis (GSVA)

GSVA (GSVA_1.36.3) refers to a non-parametric and non-supervised method of transcriptome gene enrichment. GSVA transforms the dimensionality of the gene into the dimensionality of the pathway by scoring a set of genes and evaluating their biological functions. Gene sets for the current study were acquired in the Molecular Signatures database (v7.0). Furthermore, the GSVA was used to score each gene set in aggregate to assess potential biological functional changes across samples.

### 2.8. Quantitative real-time polymerase chain reaction (qRT-PCR)

In order to extract total tissue RNA, Trizol reagent (Takara Bio, Inc.) was applied in this study. In line with the instruction of the manufacturer, the complementary (c)DNA was prepared with total RNA utilizing a HiScript II Q RT SuperMix (Vazyme Biotech, Nanjing, China). By adopting a Bio-Rad CFX96 RT-PCR detection system, we performed quantitative analysis on mRNA by adopting ChamQ Universal SYBR qPCR Master Mix (Vazyme Biotech, Nanjing, China) (Bio-Rad, CA, United States). The following thermocycling program was used: 30-s initial denaturation under 95°C; subsequent 15-s denaturation under 95°C. Next, 60-s extension under 60°C was performed for 40 cycles. Table 1 presents primer sequences. GAPDH was applied to be the endogenous reference. This study adopted 2^–ΔΔCT^ method to analyze mRNA levels (see Table 1).

**Table 1:** Primer sequences for three hub genes (5′-3′).

Gene name	Forward primer(5′-3′)	Reverse primer(5′-3′)
HTRA1	ACCGACAGGCCAAAGGAAAA	GCTCCTGAGATCACGTCTGG
P2RY6	CTTGCGTGTGAAAGCTGAGAA	ACTGCCTGGAGCATCTGGCTA
RCAN1	AGCACTTGCTTGCGGAACTC	AGTTACACGTTGCACGGTTGG
GAPDH	GCACCGTCAAGGCTGAGAAC	TGGTGAAGACGCCAGTGGA

### 2.9. Western-blotting (WB) assay

After fast dissection of cartilage tissues, the samples were treated with instantaneous homogenization on ice using phosphatase and protease inhibitor-containing RIPA buffer. After 15-min centrifugation of lysates at 12,000 × g under 4°C, we collected supernatants to quantify protein contents using the BCA assay kit (Beyotime, China). Then, proteins were denatured and isolated on 10–12% SDS-PAGE gel (30 μg per lane), followed by transfer on PVDF membranes (Millipore, United States). Additionally, we blocked membranes with skimmed milk (5%) within TBST for a 1 h period at ambient temperature. Subsequently, they were rinsed with TBST three times. Subsequently, the membranes were exposed to overnight primary antibody incubation at 4°C, including rabbit anti-HTRA1 (1:1000, Proteintech,55,011–1-AP), rabbit anti-P2Y6 (1:1000, Bioss, bs-12075R), mouse anti-GAPDH (1:5000, Proteintech, 60,004–1-Ig), as well as rabbit anti-Calcipressin1 (1:500, Proteintech, 14,869–1-AP). After washing in TBST thrice, horseradish peroxidase (HRP)-labeled secondary antibody was added to incubate membranes at ambient temperature. An enhanced chemiluminescence technique was applied to assess protein expression, while a ChemiDoc XRS system (Bio-Rad, United States) was utilized for imaging the blots. Furthermore, the blot images were quantitatively assessed with Image J (National Institutes of Health, USA), with data being normalized to GAPDH.

### 2.10. Statistical analysis

GraphPad Prism software (version 9.0, GraphPad Software, Inc., La Jolla, CA) was utilized in analyzing experimental data. A t-test was adopted for determining statistically significant data and presented as means ±SEM. *p < 0.05* stood for the statistical significance.

## 3. Results and discussion

### 3.1. Identification of OA-related DMGs

**Figure 2:**
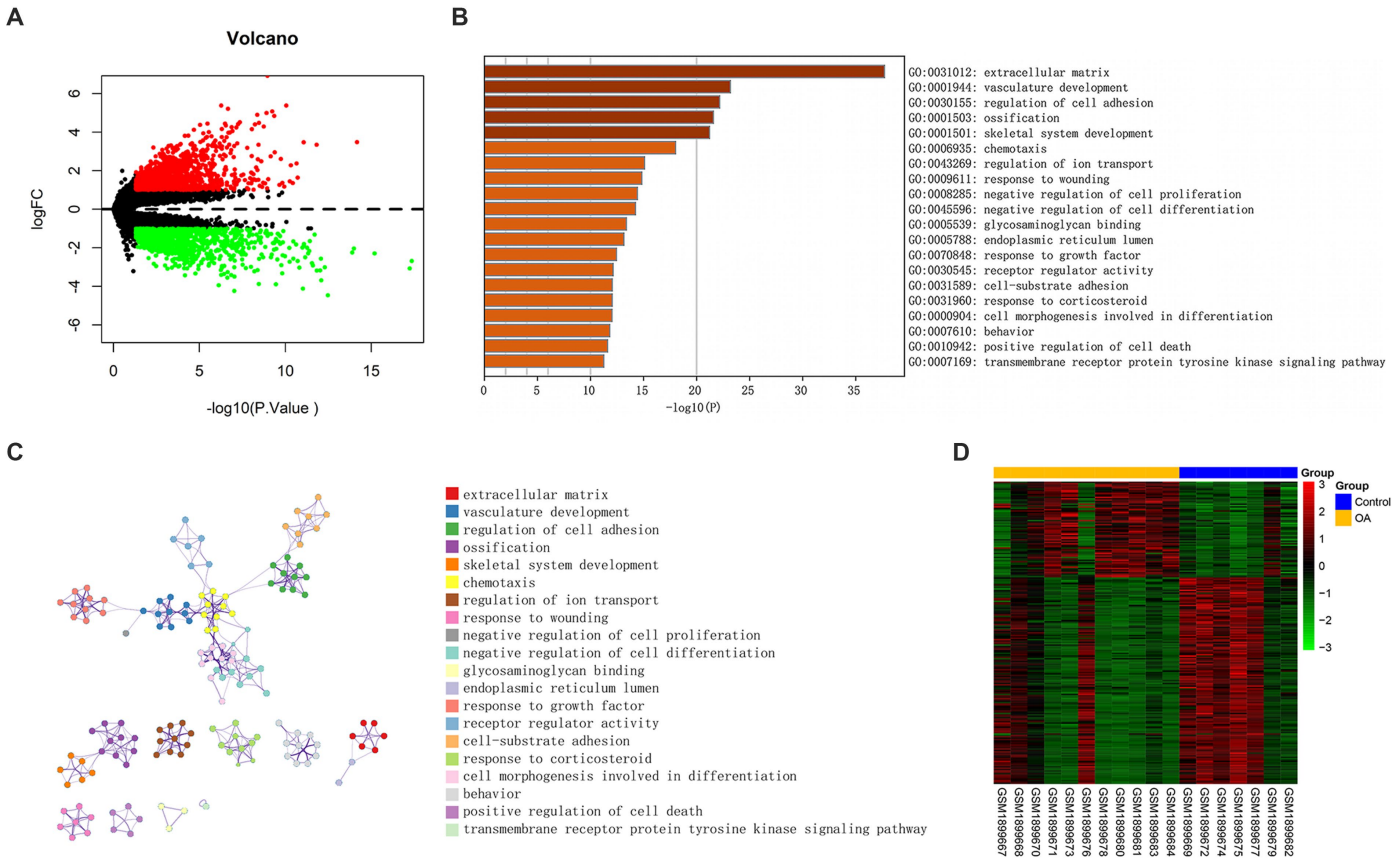
Differentially expressed genes associated with OA. **(A)** Volcano plot of DEGs between OA and control (vioplot_0.3.7). **(B)** Significantly enriched paths. **(C)** Cluster network composed of enriched paths, where nodes sharing the same cluster are usually close to one another. **(D)** A heatmap of DMGs was built with data from GSE73626. The *β* value of one group was <0.2, but the other one was required to be >0.3, and *p* < 0.05.

**Figure 3:**
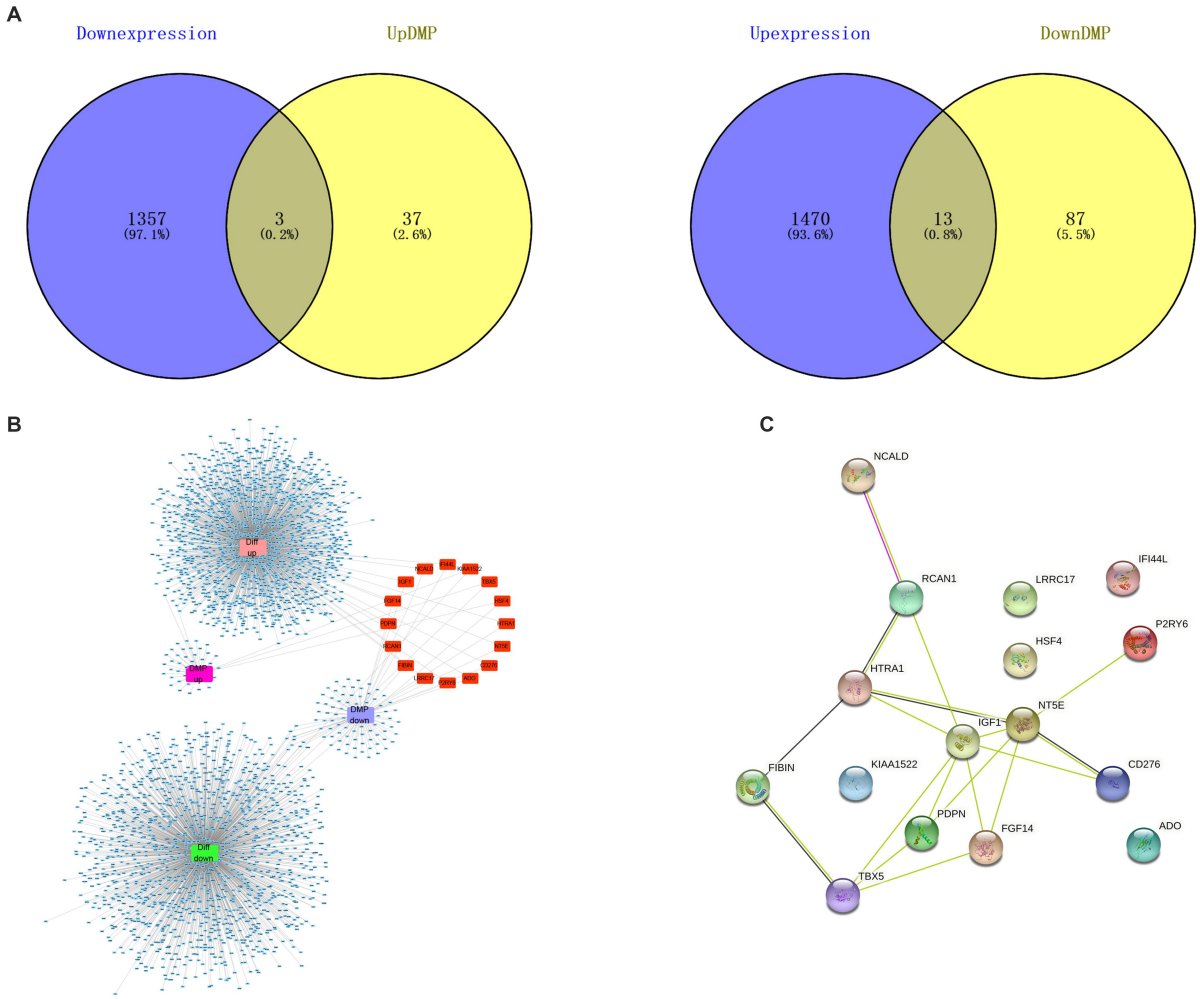
Differentially methylated differentially expressed genes associated with OA. **(A)** Thirteen hypomethylated and up-regulated genes and 3 hypermethylated and down-regulated genes were displayed in Venn diagram. **(B)** Interaction network of high expression with low methylation, and low expression with high methylation (red part). **(C)** PPI networks of 16 genes showing high methylation-low expression together with low methylation-high expression showed the mutual interaction among these genes (stringr_1.4.1).

On the basis of NCBI GEO, transcriptome data from 38 samples were obtained in GSE114007. The differential expression profile screening was performed as follows: |log FC| > 1 and *p* < 0.05. Altogether, 2,843 DEGs comprising 1,483 with up-regulation were detected, while 1,360 with down-regulation were detected (Figure 2A). GO, as well as KEGG analyzes of DEG, indicated those enriched pathways related to the extracellular matrix, the development of the vasculature, the regulation of cell adhesion, ossification, the development of the skeletal system, and other signal mechanisms. Different genes associated with these pathways could be related to OA (Figures 2B,C). In addition, 18 differentially methylated groups of the 450 K data set from GSE73626 of the NCBI GEO public database were investigated using the ChAMP package and 221 differentially methylated probes consisting of 151 down-regulated and 70 up-regulated probes (Figure 2D). Furthermore, 3 hypermethylated and down-expressed genes, including *KIAA1522*, *HSF4*, and *TBX5*, and 13 hypomethylated and up-expressed genes, including *HTRA1*, *P2RY6*, *NT5E*, *IGF1*, *RCAN1*, *CD276*, *NCALD*, *IFI44L*, *LRRC17*, *FIBIN*, *ADO*, *PDPN*, and *FGF14* were selected, respectively (Figure 3A). There were interactions among these genes (Figures 3B,C).

### 3.2. WGCNA and hub modules identification

**Figure 4:**
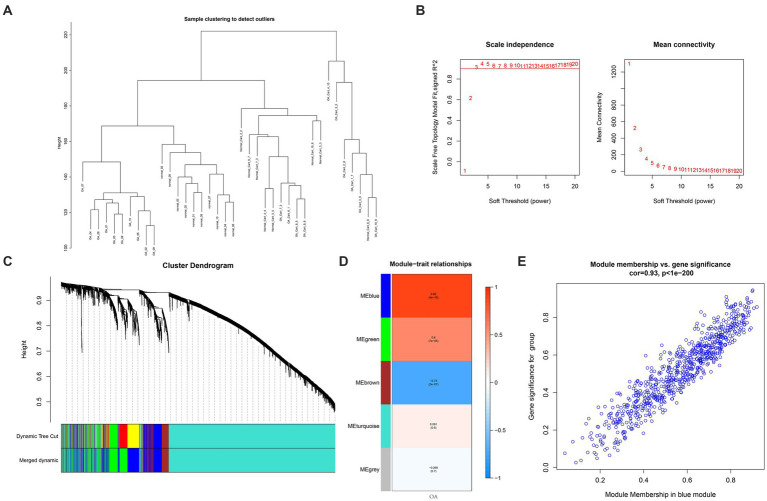
WGCNA. **(A)** Clustering dendrogram showing 5,000 samples. **(B)** Soft-threshold power in WGCNA. **(C)** A method for hierarchical clustering to construct a clustering tree structure for TOM matrices to estimate network connectivity. **(D)** Heatmap showing association of ME with OA modules. **(E)** A scatterplot relating the GS of the group to MM within blue module. GS and MM showed a significant correlation, and 753 essential genes were identified.

For investigating disease modules associated with OA, WCGNA was applied to the genes of the GSE114007 dataset with a top 5,000 variance. No outliers were found in the sample clustering (Figure 4A). This work analyzed soft-threshold power *β* using “sft$powerEstimate” function which was set at 3 (Figure 4B). A total of 5 gene modules, including blue (*n* = 753), brown (*n* = 354), green (*n* = 548), gray (*n* = 1), and turquoise (*n* = 3,344), were discovered by the TOM matrix (Figure 4C). In WGCNA’s clustering tree, each leaf node represents a gene, and the clustering process starts at the bottom leaf node and gradually merges into higher-level module nodes. The distance between leaf nodes represents the gene expression similarity between two different genes. A closer examination of the relationships between modules and phenotypes revealed the strongest phenotypic link (cor = 0.92, *p* = 4e-16) between the blue module (MEBlue) and the OA (Figure 4D). In Figure 4E, the abscissa displayed the connection between the blue module and the gene expression profile, while the coordinate showed the relationship between the OA group and the blue module. We further validated a significant association of gene significance (GS) with module membership (MM) within blue module by analyzing two coordinates and identified 753 essential genes in this module (Figure 4E).

### 3.3. Identification of hub genes for OA

**Figure 5:**
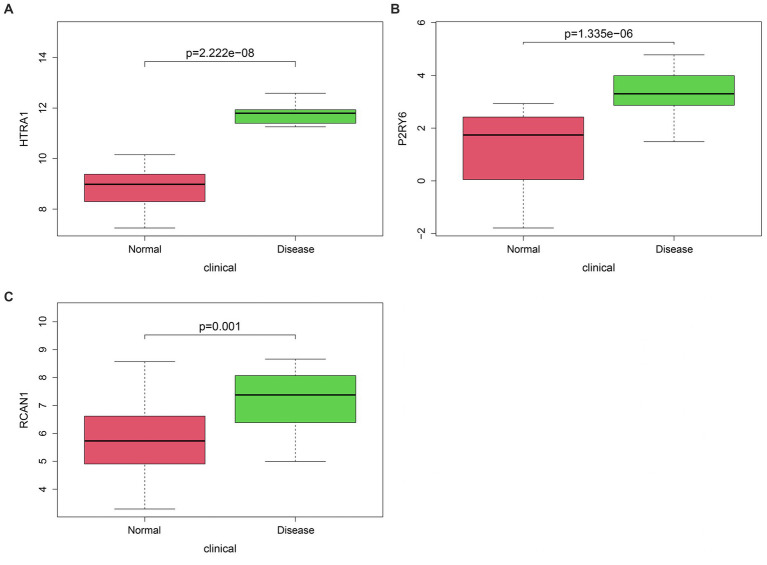
Classification of hub genes **(A–C)**. The difference in HTRA1 (*p* = 2.222e-08), P2RY6 (*p* = 1.335e-06), and RCAN1 (*p* = 0.001) levels could be observed in OA compared with control groups. HTRA1, HtrA serine peptidase 1; P2RY6, pyrimidinergic receptor P2Y6; RCAN1, regulator of calcineurin 1.

Three hub genes for OA (*HTRA1*, HtrA serine peptidase 1; *P2RY6*, pyrimidinergic receptor P2Y6 and *RCAN1*, regulator of calcineurin 1) were identified by intersecting WGCNA blue module genes with differentially methylated differentially expressed genes (Figure 5).

### 3.4. Identification of the relationship between hub genes and immune cells

**Figure 6:**
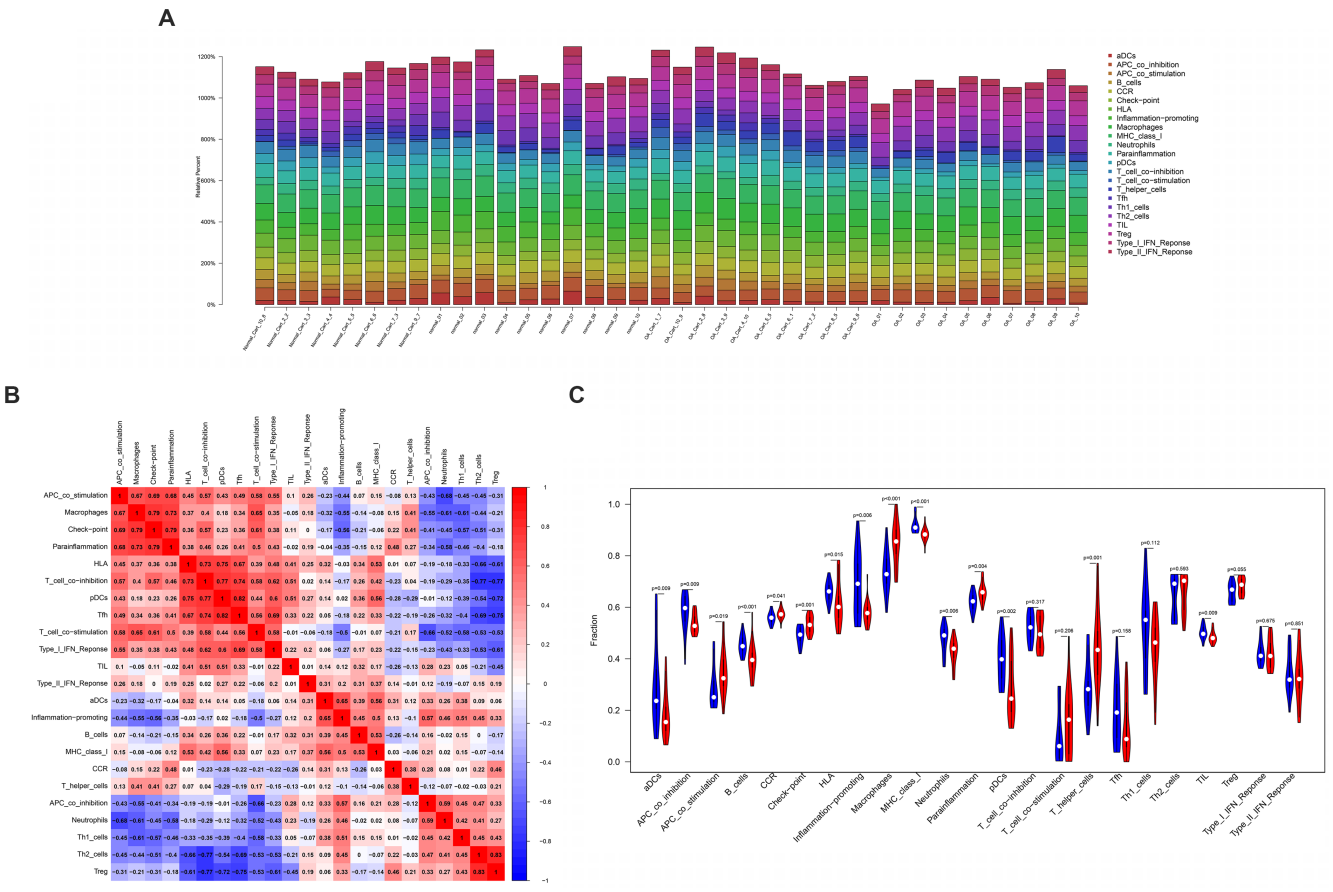
The interaction between immune cells. **(A)** Bar graph of various types of immune cells in a single sample. **(B)** Heatmap of immune cell correlation. **(C)** Vioplot of immune cell proportions.

**Figure 7:**
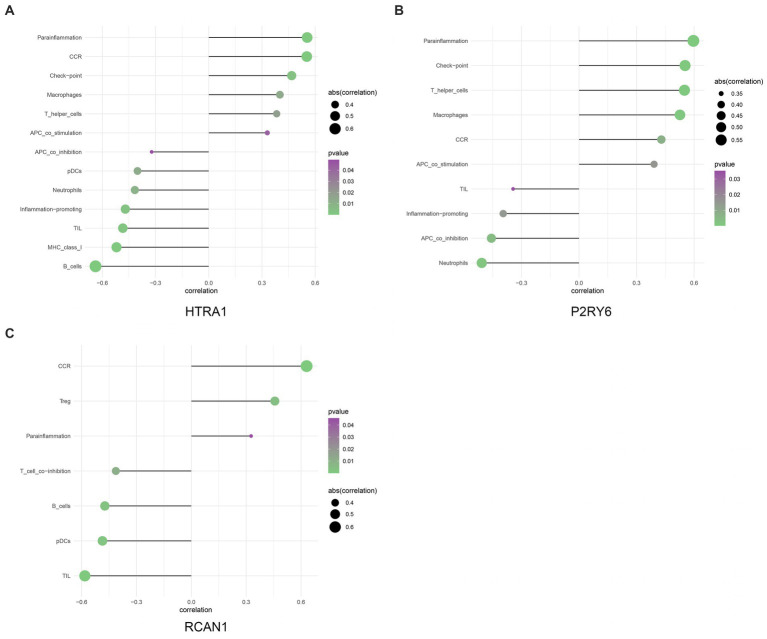
The relationship between hub genes and immune infiltration **(A–C)**.

This work applied ssGSEA to analyze the relationship of hub genes with infiltrating immune cells in all patients (Figure 6). The results demonstrated a significant positive association between *HTRA1* and para-inflammation, CCR, check-point, macrophages, T-helper cells, and APC-co-stimulation. Additionally, a significant positive relationship between *P2RY6* and para-inflammation, check-point, T-helper-cell, and macrophages was also established. Moreover, a significant negative relationship between *RCAN1* and TIL, pDCs, B-cells, and T-cell-co-inhibition was identified (Figure 7).

### 3.5. Functional annotation

**Figure 8:**
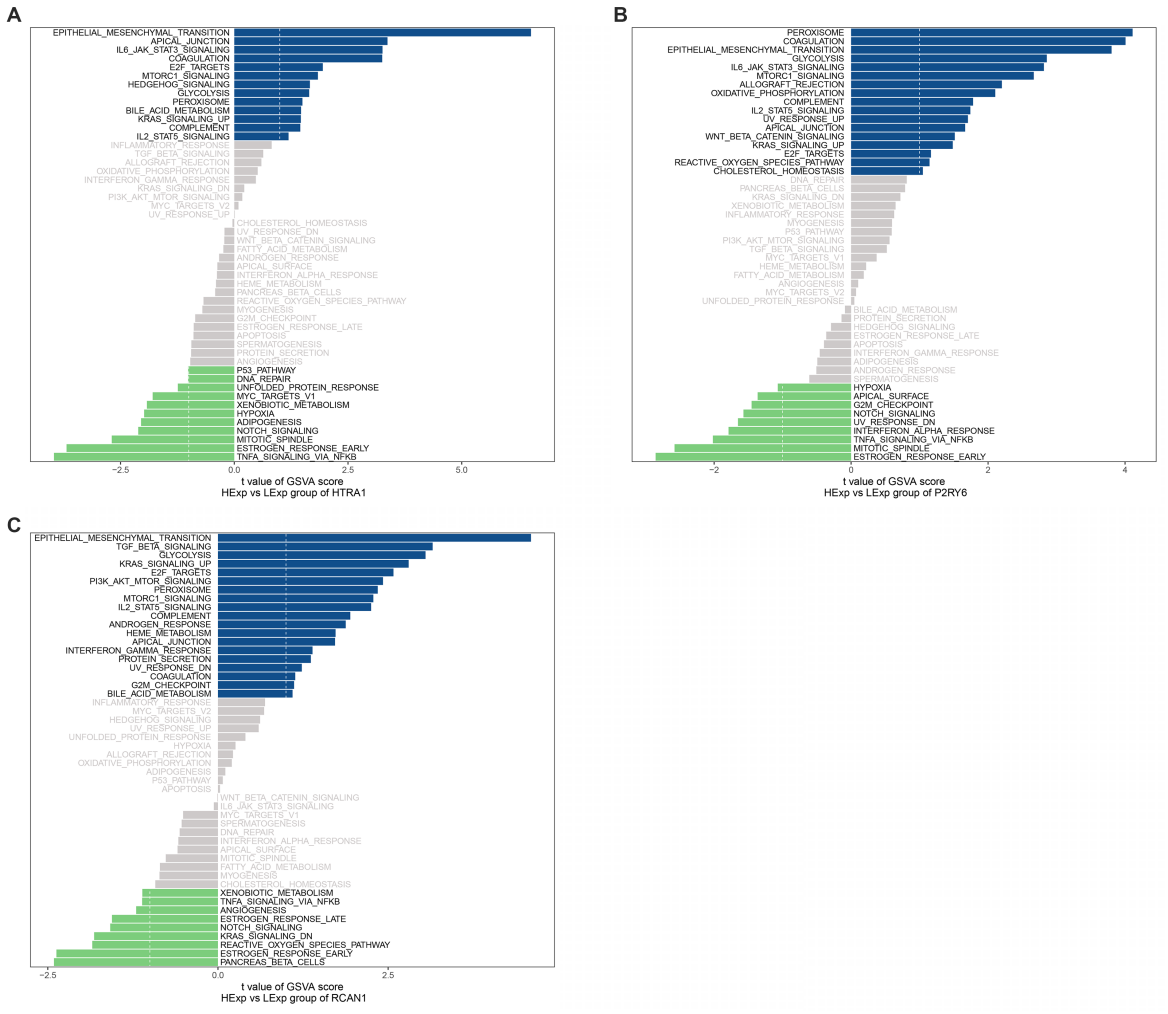
GSVA. Pathways enriched by **(A)** HTRA1; **(B)** P2RY6; and **(C)** RCAN1. A positive association is symbolized by a blue band, and a negative association is symbolized by a green band.

We use the gsva algorithm of GSVA package to score the hallmark pathway of each sample, and then use the limma package to identify the pathway with differential expression between high and low expression of hub genes. Unique signaling pathways related to the three hub genes were identified by GSVA, and their influence on disease progression was investigated. As a result, *HTRA1* up-regulation was mainly enriched into pathways like IL6-jak-stat3(interleukin 6- Janus kinase- signal transducer and activator of transcription-3), apical-junction, epithelial-mesenchymal-transition; *P2RY6* up-regulation was mainly associated with the peroxisome, coagulation, epithelial-mesenchymal-transition; and *RCAN1* up-regulation was mainly associated with epithelial-mesenchymal-transition, TGF-β-signaling (Transforming growth factor β signaling), glycolysis, and other signal pathways (Figure 8).

### 3.6. Validation of hub genes

**Figure 9:**
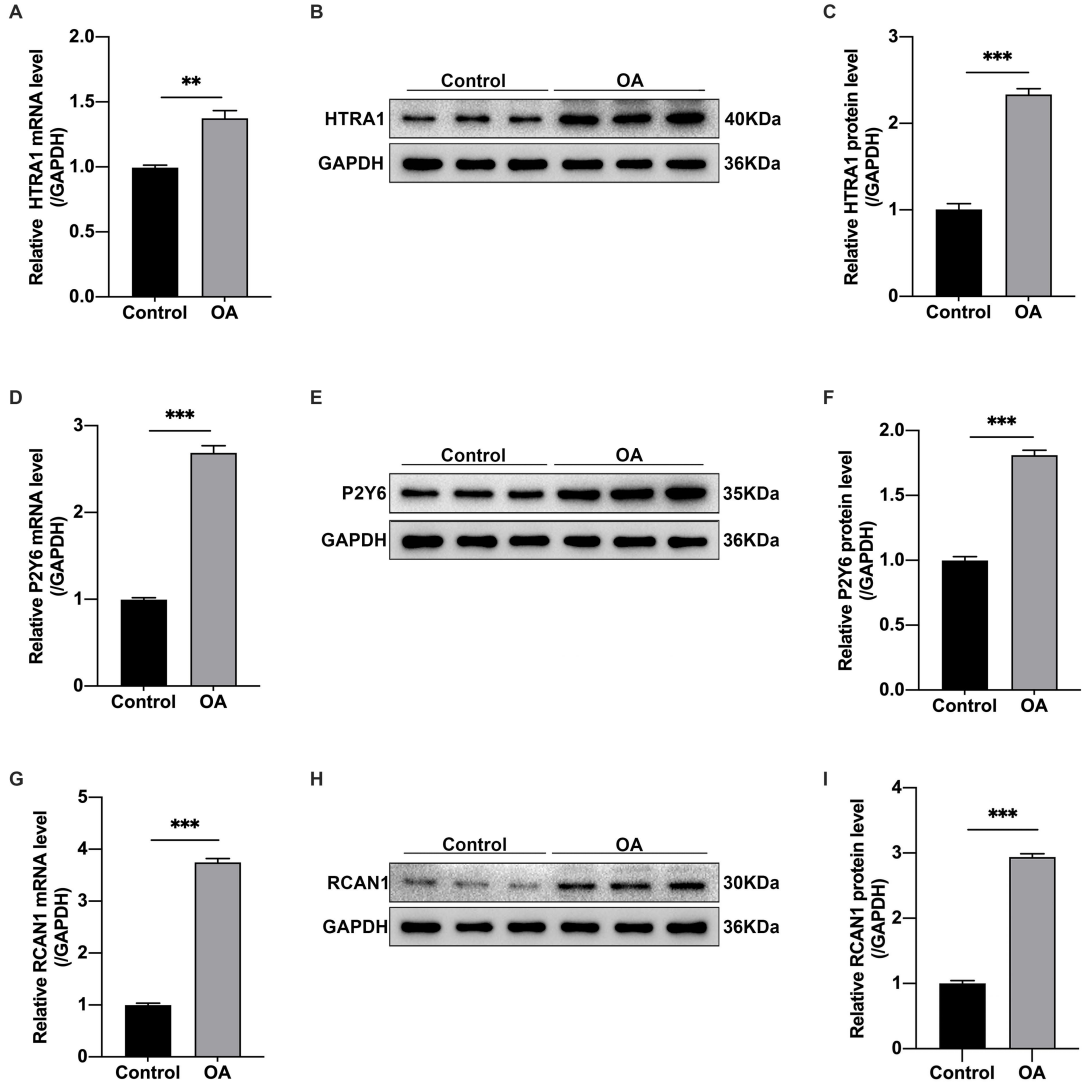
By adopting RT-qPCR, the above 3 hub genes were discovered to be differentially expressed in the OA group in relative to the control group. **(A)** HTRA1, **(D)** P2RY6, and **(G)** RCAN1. GAPDH expression was taken as standard for all samples. Means ±SEM (*n* = 9) are used to represent values, *p < 0.05, **p < 0.01, ***p < 0.001 vs. control group, OA, osteoarthritis. **(B,C,E,F,H,I)** Quantitative analysis and Western blot assay exhibiting HTRA1, P2RY6, RCAN1 protein levels, and corresponding NCs, with GAPDH being the endogenous reference. In addition, data are indicated by means ±SEM, *p < 0.05, **p < 0.01, ***p < 0.001.

To verify the above-mentioned findings, RT-qPCR and WB assays were performed. According to RT-qPCR assays, OA group had increased HTRA1, P2RY6, and RCAN1 mRNA levels in relative to control (*p < 0.01*, *p < 0.001, p < 0.001*, separately, Figures 9A,D,G). Based on the WB assay, the OA group had an increase in the expression of the HTRA1, P2RY6, and RCAN1 proteins in comparison with control (*p < 0.001*, *p < 0.001, p < 0.001*, separately, Figures 9B,C,F,H,I). All validations conformed to our analyzes.

We identified differentially methylated and differentially expressed genes by combining transcriptome analysis, methylation, and WGCNA in patients with OA and control groups. We then analyzed association of these hub genes with inflammatory pathways, for analyzing how signal transduction affected the occurrence and progression of OA. Three OA-related hub genes were identified: *HTRA1*, *P2RY6*, and *RCAN1*. In line with the prediction, we confirmed that the expressions of HTRA1, P2RY6, and RCAN1 of the control group decreased relative to the OA group. Collectively, *HTRA1*, *P2RY6*, and *RCAN1* were hub genes that were markedly related to immune cell infiltration degrees within OA.

## 4. Discussion

*HTRA1* was first discovered in human fibroblasts as the potential serine protease and was also considered L56 or serine protease 11 found on chromosome 10q26.13 (22). According to recent research, *HTRA1* was found both intracellularly and extracellularly as a secretory protein and was a microtubule-associated protein to control cell motility (23). TGF-β/BMP pathway was connected to *HTRA1* in a stage-and-dose-dependent manner for regulating bone formation and bone resorption (24, 25). *HTRA1* is the new factoring mediating arthritic disorders, which is reported to have increased expression within articular cartilage and synovial fluid among RA and OA cases (26, 27). *HTRA1* participated in the association of MMP-13, TGF-protein, and discoidin domain receptor 2 (DDR-2) (28–31). *HTRA1* showed up-regulation, which was associated with DDR-2 activation during OA development (29). Down-regulated DDR-2, the cell surface receptor, was responsible for the prevention of articular cartilage degradation (32). MMP-13 was capable of degrading the extracellular matrix, especially type II collagen and acrasin, influencing OA occurrence and development (5). TGF-β also affected OA occurrence and progression (29, 31). Inactivated TGF-β/Smad1 signal could slow the progression of OA, while an increase in TGF-β1 up-regulates HTRA1 within chondrocytes by phosphorylating Smad2/3. It promoted the hydrolytic cleavage of ECM while down-regulating type VI collagen level, thereby aggravating OA deterioration (27–29, 31). In this study, many of the predicted *HTRA1* enrichment pathways were highly related to OA initiation and development. Collectively, suppressing *HTRA1* possibly induces the weakening of OA progress. Identifying and regulating the expression of *HTRA1* will be the core of finding new therapeutic targets to prevent and treat OA.

As for genetics, the 11q13 region was reported to be involved in cancer development, as well as the *P2RY6* gene positioned in the 11q13.4 region (33). The *P2Y6* receptor, as the transcriptional product of the *P2RY6* gene which represents the G protein-coupled receptor of P2 receptor family, can be activated through extracellular nucleotides. *P2Y6* receptors are found in virtually all tissues and are associated with various reactions, such as inflammatory responses in the airways. *P2Y6* shows prominent expression within infiltrating T lymphocytes of inflammatory bowel disease (IBD) patients, breast cancer (BC) tissues and intestinal epithelial cells (34, 35), virus-infected macrophages (36), epithelial cells and fibroblasts during allergic inflammation and pulmonary fibrosis, together with CD31+ endothelial cells among atherosclerosis patients (37, 38). The *P2Y6* receptors show wide expression in the lung, heart, spleen, intestine, aorta, placenta, and some brain regions (39). *P2Y6* receptor activation is related to vascular inflammatory response together with atherosclerotic lesion occurrence by improving macrophages’ pro-inflammatory response (40, 41). We predicted the interaction between *P2RY6* and immune cells. *P2RY6* expression was significantly positively related to para-inflammation, check-point, T-helper-cell, and macrophages, and a negative correlation with neutrophils, APC-co-inhibition, and TIL. The relationship between osteoarthritis and immune cells is well known. It is possible to control OA initiation and progression by modulating *P2RY6* expression and then impacting immune cell infiltration. In addition, the activation of strong fibrogenic factors and a variety of inflammatory reactions are closely related to joint stiffness and pain. According to various studies, synovitis, as well as the immune system, play a significant part in the OA bone marrow lesions, which impact the entire joint (42).

*RCAN1*, previously known as ADAPT78, DSCR1, or MCIP1, was found on chromosome 21 q22.12 region, which contained 7 exons, including three (exons 5, 6, and 7) showing consistency within each *RCAN1* subtype. Until now, at least 3 *RCAN1* protein and mRNA subtypes are identified within different living bodies: *RCAN1.1S* (*RCAN1 subtype 1 short*), *RCAN1.1 L* (*RCAN1 subtype 1 long*), as well as *RCAN1.4* (*RCAN1 subtype 4*) (43). The *RCAN1.1* and *RCAN1.4* subtypes were reported to reduce CnA activity (44), where CaN was necessary to activate transcription and induce cell hypertrophy with different cells (45, 46). TGF-β performed two important functions: inducing cell hypertrophy and regulating the accumulation of extracellular matrix. Furthermore, a study supported the potential role of CnA in TGF-β signal transduction (47). Interestingly, TGF-β/Smad2,3 signal transduction was beneficial for the maintenance of chondrocyte homeostasis, while the TGF-β/Smad1,5,8 signal pathway can cause chondrocyte hypertrophy. Various studies have also reported the close association of TGF-β/Smad pathway with OA initiation and development. Simultaneously, the knockdown of *RCAN1.4* cells was shown to accelerate osteoclast formation (48). Furthermore, chondrocyte hypertrophy, extracellular matrix remodeling, and abnormal subchondral osteogenesis were the main manifestations of primary osteoarthritis. It was also verified that regulating TGF- β/Smad RNA and protein levels might affect epithelial-mesenchymal transition (49). Therefore, the interaction between the two pathways was certain, and these two pathways were highly enriched by *RCAN1* as our results. So *RCAN1* was worthy of being the new research direction for OA development.

The advancement of Genomics technology has led to the identification of key genes as a crucial aspect of research. Presently, various methods with high utilization rates include: (1) bioinformatics-based screening methods, which identify genes associated with diseases through analysis of genomic, transcriptomic and proteomic data. A large number of candidate genes can be screened quickly, (2) Gene expression-based screening method, by comparing the gene expression profiles of diseased and normal tissues, to identify disease-related genes. It can directly reflect the expression of genes in the organism, but requires a large number of samples and the support of high-throughput sequencing technology, (3) Gene editing-based screening methods verify whether a gene is associated with a disease by performing operations such as knocking out, overexpression or mutation of the gene. The association of a gene with a disease can be directly demonstrated, but candidate genes need to be identified first, and (4) Genetics-based screening methods that identify genes associated with disease through genetic analysis of family lines, populations, etc. The genetic relationship between a gene and a disease can be directly demonstrated. By using proteomics, Luo et al. (50) found that Astragaloside IV(ASG-IV)plays a key role in apoptosis of OA chondrocytes. According to Liu et al. (51), 71 differentially expressed circRNAs are involved in OA, and CircRNA-CER functions as a spring for miR-136 to regulate MMP13 expression. Thus inducing extracelluar matrix (ECM) degradation. Different screening methods have their own advantages and disadvantages and the appropriate method needs to be chosen according to the specific situation. In this study, bioinformatics analysis technology was used, which has the advantage of being able to analyze a large number of genetic data simultaneously, thus improving the efficiency and accuracy of gene screening. In addition, bioinformatics analysis technology can help to identify new genetic variants and mutations, leading to a better understanding of disease mechanisms and therapeutic approaches.

## 5. Limitation

The current study has some limitations. First, it is difficult to collect normal knee cartilage specimens clinically, which is contrary to the clinical concept of limb salvage; thus, we can only select patients with major traffic accidents or injuries who need thigh amputation for postoperative sampling. Therefore, only 3 of normal knee cartilage and 6 of hip cartilage were collected and compared with 9 samples of knee cartilage from OA patients. This insufficient sample size and the difference in cartilage in different positions may lead to a deviation in experimental results. Second, the concentration of mRNA extracted from the samples using the qRT-PCR technique was unstable. Gene expression might have caused this deviation. Thirdly, this study did not consider the impact of race on gene expression and mutations, and cannot clarify whether data from foreign databases have bias on domestic patients. In order to overcome the limitations of this experiment, we will continue to collect tissues from severely injured patients who require amputation, and then determine the differences in gene expression and methylation levels in knee cartilage samples, and collect domestic patient data for analysis.

## 6. Conclusion

In summary, we conducted a joint analysis of the transcriptome, methylation, and WGCNA, and found three new hub hypomethylated and up-regulated genes (*HTRA1*, *P2RY6*, and *RCAN1*), which were highly expressed in the OA group. These findings were further validated through RT-qPCR and WB assays. As suggested by subsequent investigation, the peroxisome, epithelial-mesenchymal transition, and TGF-β pathways are connected to these three hub genes, which may be related to OA initiation. Additional research into the mechanisms associated with these hub genes may offer innovative treatments for the disease.

## Data availability statement

The original contributions presented in the study are included in the article/supplementary material, further inquiries can be directed to the corresponding author.

## Ethics statement

The studies involving human participants were reviewed and approved by our study protocols gained approval from the First Affiliated Hospital of Dalian Medical University (PJ-KS-KY-2022–236). The patients/participants provided their written informed consent to participate in this study.

## Author contributions

Z-CC was in charge of study concept and design. XT provided administrative assistance. J-YZ and Z-YL provided human or material samples. Z-CC was responsible for analyzing data. Z-CC, JZ, and YG were in charge of analyzing and interpreting data. Z-CC was in charge of writing this manuscript. All authors contributed to the article and approved the submitted version.

## Conflict of interest

The authors declare that the research was conducted in the absence of any commercial or financial relationships that could be construed as a potential conflict of interest.

## Publisher’s note

All claims expressed in this article are solely those of the authors and do not necessarily represent those of their affiliated organizations, or those of the publisher, the editors and the reviewers. Any product that may be evaluated in this article, or claim that may be made by its manufacturer, is not guaranteed or endorsed by the publisher.

## Footnotes

^1^www.metascape.org
